# Role of Antioxidant Vitamins and Other Micronutrients on Regulations of Specific Genes and Signaling Pathways in the Prevention and Treatment of Cancer

**DOI:** 10.3390/ijms24076092

**Published:** 2023-03-23

**Authors:** Oladapo F. Fagbohun, Caroline R. Gillies, Kieran P. J. Murphy, H. P. Vasantha Rupasinghe

**Affiliations:** 1 Department Plant, Food, and Environmental Sciences, Faculty of Agriculture, Dalhousie University, Truro, NS B2N 2R8, Canada; fg283429@dal.ca; 2 Department of Animal Science and Aquaculture, Faculty of Agriculture, Dalhousie University, Truro, NS B2N 2R8, Canada; caroline.gillies@dal.ca; 3 Department of Medical Imaging, Faculty of Medicine, University of Toronto, Toronto, ON M5T 2S8, Canada; kieran.murphy@uhn.ca; 4 Department of Pathology, Faculty of Medicine, Dalhousie University, Halifax, NS B3H 4R2, Canada

**Keywords:** antioxidants, cancer, nutrigenomics, vitamins, minerals, cell signaling, genes

## Abstract

Cancer is an escalating global issue, with 19.3 million new cases and 9.9 million deaths in 2020. Therefore, effective approaches to prevent cancer are urgently required. Diet plays a significant role in determining cancer risk. Nutrients and food bioactives influence specific signaling pathways in the body. Recently, there have been significant advances in cancer prevention research through nutrigenomics or with the effects of dietary components on the genome. Google Scholar, PubMed, and Scopus databases were used to search for peer-reviewed articles between 2017 and 2023. Criteria used were vitamins, minerals, tumors, cancer, genes, inflammation, signaling pathways, and nutrigenomics. Among the total of 1857 articles available, the highest relevant 90 articles that specifically discussed signaling pathways and genes on cancer cell lines and human cancer patients were selected and reviewed. Food sources are rich in antioxidant micronutrients, which are effective in activating or regulating signaling pathways involved in pathogenesis and cancer therapy by activating enzymes such as mitogen-activated protein kinase (MAPK), protein kinase C (PKC), and phosphatidylinositol 3-kinase (PI3K). The micronutrients are involved in the regulation of β-catenin (WNT/β-catenin) including mutations in Kras and epidermal growth factor receptor (EGFR) alongside inhibition of the NF-kB pathway. The most common mechanism of cancer prevention by these micronutrients is their antioxidative, anti-inflammation, and anti-apoptosis effects. This review discusses how nutrigenomics is essential and beneficial for developing cancer prevention and treatment approaches.

## 1. Introduction

Cancer is the uncontrollable division of cells in the body due to damage to genetic material. The prognosis of cancer depends on the type of cancer and whether the cancer is malignant (spreading to other tissues) or benign (contained to a single tissue type). In 1893, Rudolph Virchow was the first to identify that inflammation is associated with the development of cancer. Research on cancer progressed significantly throughout the 1970s, where there were many advances, namely the development of antineoplastics agents that target the tumour microenvironment [1]. In 1989, vascular endothelial growth factor (VEGF) was identified, allowing for more targeted antineoplastics that have fewer adverse effects than traditional antineoplastics. Advancements in cancer prevention and treatment methods as well as cancer screening to diagnose cancer earlier, are being continued worldwide [2,3].

Cancer is estimated to account for 28.2% of deaths in 2021, making it the leading cause of death in Canada [3]. There appears to be an increased mortality rate in the last two years as a result of complications arising from the COVID-19 pandemic. Moreover, many of these complications were attributed to underlying diseases other than cancer, such as diabetes and cardiovascular diseases, which are metabolic diseases. The Canadian Cancer Society predicts that in 2021 and many years to come, two in five Canadians will be diagnosed with any type of cancer, and one in four Canadians will die due to cancer. The most common types of cancer are lung, breast, colorectal, and prostate cancers [3,4]. These statistics suggest the need for urgent cancer prevention methods. One important and easily adaptable approach is meeting the recommended micronutrient requirement and applying nutrigenomics tools to make the above more efficient and personalized. The main micronutrients with identified cancer-prevention properties are vitamin A, vitamin C, vitamin D, vitamin B_9_ (folate), selenium, zinc, and magnesium [5,6,7,8,9,10,11,12,13,14,15]. Research in this discipline has supported the annual decline in cancer incidence and mortality [3,16]. Therefore, this review discusses the specific roles of various antioxidant micronutrients in cancer therapy and presents the most recent research on cancer treatment and prevention.

## 2. Nutrigenomics in Cancer Therapy

**Figure 1:**
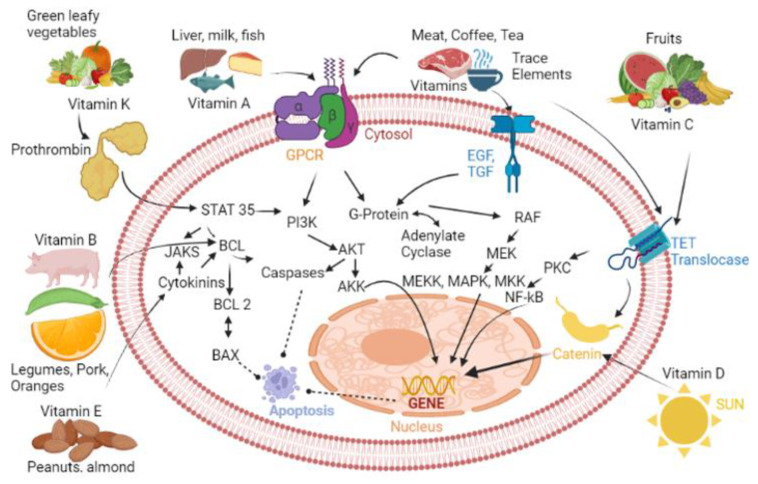
Cancer-associated cell signaling pathways that can be regulated by selected vitamins and minerals. List of abbreviations: GPCR, G-protein-coupled receptor; EGF, Epidermal growth factor; TGF, Transforming growth factor; NF-κB, Nuclear Factor kappa B; AKT, Serine/threonine kinase; MAPK, Mitogen-activated protein kinase; MEKK, MAPK/EKK kinase kinase; MKK, MAPK Kinase; PKC, Protein Kinase C; PI3K, Phosphatidylinositol-3-kinase; BCL, B-cell lymphoma protein; AKK, Serine/threonine kinase 2; JAKS, Janus kinase.

Nutrigenomics is the study of how nutrients change gene expression by having a direct effect on the genome or indirectly by regulating biological mechanisms [10,12,17,18]. This field of study began after the completion of the Human Genome Project in 2003 [10,12,16]. Understanding the nutrigenomics of nutrients can allow for personalized nutrition therapies to be used in the prognosis, treatment, and prevention of cancer on the basis of which nutrients have a protective effect on the body [10]. Nutrients affect gene regulation, which impacts epigenomic, transcriptomic, proteomic, or metabolomic pathways [17,18]. Each nutrient affects these pathways in different ways, resulting in different effects [18,19]. Further investigation of nutrigenomic aspects of cancer can aid in the prevention and treatment of cancer. The diet–gene interactions can be characterized to determine which nutrients impact specific genes of biochemical pathways, and how those genes can initiate cancer cell formation (Figure 1).

In order to understand the application of nutrigenomics as a control tool for cancer therapy, especially by caregivers and health workers, it is essential we familiarise ourselves with postgenomic and posttranscriptional events from single cells all the way to whole behaviors. The advent of genomics in food aspects is undoubtedly shifting our views of humans and informing the next generation of disease care as well as caregivers. The food strategy of ensuring adequate essential nutrient intake in a diverse population effectively equates to nutrient overdose and thus relies on the capacity of human physiology to appropriately regulate each essential nutrient across a wide range of dietary intakes [16]. Strategically, combating cancer using nutrigenomics involves nutritionists and dieticians who will determine and/or personalize nutrition for each individual by guided predictive knowledge of their diets. To achieve this, science will extend from new accurate and predictive measures of health based on genes, microbiota, transcripts, proteins, and metabolites [20]. This arises as a result of the different needs of individuals physiologically, genetically, and metabolically with extension to age, lifestyle, and diets [21]. This review aims to understand the role of antioxidant micronutrients (vitamins and trace elements), the core signaling pathways that they regulate, and their relationship to nutrigenomics. This information can assist caregivers, physicians, dieticians, and nutritionists in working closely with cancer patients to identify the appropriate diets for nutrigenomics-based management of cancer. Therefore, this review intends to provide insights and perspectives on the effects of nutrigenomics in cancer therapy.

## 3. Micronutrients in the Prevention of Cancer

Ancient civilizations frequently used minerals as treatments for disease, amulets, and talismans. The discovery of vitamins began in the late 19th century. It was observed that the health of people around the world was similar despite eating different foods; therefore, it was determined that the components of food contributed to heath. A variety of food, such as meat, seafood, grains, fruits, and vegetables, is required to obtain all the necessary micronutrients in the diet (Table 1). There are multiple diseases that are associated with deficiencies of micronutrients in the diets, such as scurvy, rickets, and pellagra [22]. The Vitamin Theory observed that animals fed a synthetic diet containing the known nutrients (macromolecules and salts) would not survive; therefore, other components in food contribute to survival. Casimir Funk coined the word “vital amines”, which was later shortened to “vitamin”. He proposed four vitamins: anti-beriberi, anti-rickets, anti-scurvy, and anti-pellagra. Later, Elmer McCollum and his assistant Marguerite Davis found two vitamins called fat-soluble A and water-soluble B. As vitamin research continued, all the vitamins were discovered, as well as provitamins (such as β-carotene), and their function was determined. It took 50 years to determine the structures and be able to synthesize all vitamins [17,23]. Overall, the continued research of biology, anatomy, physiology, and pharmacology has improved drug treatments and disease therapies.

Research on the medicinal uses and health benefits of minerals continued throughout the Middle Ages and continues today in the form of medical geology, which is a new field of study that focuses on the effects of minerals on the body. Malachite, a compound containing copper, was often used to treat wounds. Medical geology is a new field of study that focuses on the effect of minerals on the body [18].

### 3.1. Vitamin A and Carotenes

Vitamin A, or retinol, is present in animal products such as liver and eggs [11]. Carotenes, the precursors of vitamin A, are often found in plant-based foods [10,11]. For example, lycopene is a type of carotene that provides red color in tomatoes, and it is particularly important in the prevention of prostate cancer. Vitamin A is effective at reducing the risk of gliomas, lung cancer, colorectal cancer, and breast cancer [10,11]. As an antioxidant, vitamin A prevents DNA damage due to reactive oxygen species (ROS), which contributes to carcinogenesis [6,10,15]. The vitamin A metabolite retinoic acid amide inhibits the Janus kinase signal transducer and activator of transcription (JAK-STAT) pathway, which prevents lung cancer by promoting apoptosis of pre-cancerous cells [10]. A study by Hu [34], using a case–control study to recruit 256 confirmed non-small cell lung cancer (NSCLC) patients, identified the association of vitamin A precursor protein with several types of cancer. The protein known as retinol-binding protein 4 (RBP4) was found to be relatively correlated to the risk of the growth of NSCLC. RBP4 families of protein are secretory molecules that bind and transport retinol from food sources after their release into the bloodstream as well as thyroxine carrier proteins to form retinol-RBP4 ternary complex for specific physiological functions. RBP4 specifically signals pro-oncogenic pathways by stimulating retinoic acid 6, which triggers their downstream activation. Furthermore, RBP4 also mediates the onset of insulin resistance. The association between vitamins and the cadence of lung cancer was studied among 38,207 men and 41,498 women in a cohort study involving 3.98 mg and 7.80 mg retinol, respectively [2]. There was an association between retinol and the risk of lung cancer, especially for men with small cell carcinomas. Therefore, the required and recommended dose needs to be implemented in dietary guidelines if vitamin A is to reduce the risk of several types of cancer.

Other uses of vitamin A involve immune functions with regulatory roles in cellular and humoral immune response [35], with the protection of epithelium integrity as the utmost priority. It also has effects on the RARs genes, which code for aminoacyl-tRNA synthetase; the RXRs genes, which code for retinoid X receptors; and the JNK genes, which code for c-Jun N-terminal kinases [10,15,36]. RARs are nuclear retinoic acid receptors involved in controlling the expression of specific subsets of genes in a ligand-dependent manner by binding specific response elements via a network of interactions with co-regulatory protein complexes directed by C-terminal ligand domain located within them as well as transrepressing other gene pathways alongside their involvement in the activation of translation, cellular differentiation, proliferation, and apoptosis of kinase cascades [37]; JNK, on the other hand, is known as c-Jun NH_2_-terminal kinase responsible for phosphorylating c-Jun at Ser-63 and Ser-73 and acts specifically since its discovery over 25 years ago as a tumor suppressor [36]. JNK is activated in response to stress and proinflammatory cytokines, such as IL-6 and TNFα, as well as mediating oncogenic transformation. This was discovered after analysis of JNK deficiency in mouse models suggested the correlation between loss-of-function mutations in the mkk-4 gene with aggressive tumor development and metastasis in human cancer [38]. Consequently, modulation of these genes has an impact on the function of these proteins resulting in changes in apoptosis, cell differentiation, and immune response [6,10].

### 3.2. Vitamin C

Vitamin C, also known as ascorbic acid, has cancer-preventing properties depending on the dose provided [10,12]. It can be found in many fruits and vegetables, such as citrus fruits, peppers, and broccoli [10]. For many decades, the role of ascorbate as an anticancer agent has been debated. Moreover, the unregulated use of vitamin C as a dietary supplement or pharmacologically applied intravenous infusion by cancer patients, with numerous reports of clinical benefits, has made it difficult to postulate authenticity. However, the lack of understanding of the mechanism of action has hindered the design of appropriate clinical trials. Vitamin C promotes apoptosis of pre-carcinogenic and carcinogenic cells at high doses but promotes cell differentiation of pre-carcinogenic and carcinogenic cells at low doses [10]. Several studies have linked the significant hydrogen peroxide production by the auto-oxidation of supra-physiological concentrations of ascorbate and stimulation of the 2-oxoglutarate-dependent dioxygenase family of enzymes with a co-factor requirement for ascorbate. Additionally, vitamin C acts as an antioxidant by reducing ROS to hydrogen peroxide. At high doses, hydrogen peroxide can accumulate in carcinogenic cells and cause apoptosis [10]. In an in vivo study carried out on laryngeal squamous cell carcinoma human subjects, vitamin C was shown to activate necrotic cell death mechanisms through ROS production as well as the stimulation of protein kinase C (PKC) signaling, thereby increasing cytosolic calcium and the reduction of the risks of malignancies [6,10].

The major precursor of vitamin C (ascorbate) potentially generates hydrogen peroxide, leading to oxidative stress, and thereby targeting cancer cells [39]. Dietary vitamin C, typically in low doses, can also cause apoptosis by acting on the Bcl-2 gene, a gene that codes for a protein that is anti-apoptotic and prevents the formation of N-nitrosamine carcinogenic compounds, causing an increased immune response [6,10]. Overall, high-dose vitamin C supplementation may be effective in preventing solid tumors and malignancies, but dietary vitamin C alone is insufficient in the prevention of cancer [10]. Recent data suggest ascorbate may have a promising role in the regulation of ten-eleven translocase (TET) DNA methylases, a major factor in tumor survival, angiogenesis, stem cell phenotype, and metastasis. Since it is highly soluble in water, it is readily acquired and distributed with constant turnover. More recent studies link the mechanism of action in cancer regulation to increased cell cycle arrest, p53 upregulation, decreased ATP levels, compromised mitochondrial function, suppression of antioxidant gene expression of Nrf-2, or cell death by apoptosis.

Although, the similar structure of dehydroascorbate and glucose means it can be taken up into the cells via GLUT transporters and then reduced by either GSH, NADH, or NADPH-dependent enzymes, thus exhausting the cell of necessary molecules and, hence, upregulation of GLUT1 in KRAS and BRAF mutant cells to account for the antitumor activity of vitamin C in colorectal cancer. Given its lack of toxicity, that it is readily available, and its low cost, vitamin C is a potential cancer-preventive agent. However, a robust clinical trial is needed to ascertain its potency.

### 3.3. Vitamin D

Vitamin D can be obtained from fish, dairy, eggs, and mushrooms, or synthesized in the skin from cholesterol in the presence of sunlight [11]. It is important for maintaining the metabolism of minerals, primarily calcium and phosphorus, in the intestine, kidneys, and bones [9,11]. This lipid-soluble vitamin participates in all proliferation, apoptosis, differentiation, metastasis, and angiogenesis [8]. Liu and colleagues [8] showed the relationship between vitamin D and lowering the risk of lung cancer as well as breast cancer and its better prognosis. The study included 813,801 human subjects from different environments in Europe and the nutrigenetic effect of vitamin D was clearly studied by determining the various signaling pathways involved in mutation to K-Ras and epidermal growth factor receptors as well as proteins involved in metastasis and proliferation of cancers such as the dysregulation of Wnt/β-catenin. Since vitamin D is synthesized by the skin and tightly regulated by sunlight exposure, lack of exposure has been discovered to increase the risk of the development of many deadly cancers. However, it is estimated that there is about 30–50% reduction in the risk of breast, colorectal, and prostate cancer by either increasing sunlight exposure or vitamin D intake to about 1000 IU/d [40]. After synthesis, it is hydroxylated in the liver to 25-hydroxyvitamin D (25(OH)D), the major form of vitamin D. Several studies suggested that the relationship between vitamin D and protection against breast cancer [41].

The mechanism by which vitamin D reduces cancer risks has been attributed to the inhibition of cancer-promoting signaling pathways, including mutations in epidermal growth factor receptor (EGFR) and the dysregulation of Wnt/β-catenin, which determines proliferation and metastasis [8,42]. Furthermore, vitamin D exerts its cancer-prevention effects by upregulating the secretion of E-cadherin and catenin, which aids in cell–cell adherence to prevent metastases and repress the expression of cyclooxygenase 2 (COX2), thereby inhibiting prostaglandin synthesis, which can stimulate tumor cell proliferation and angiogenesis. Vitamin D deficiency is associated with an increased risk of oral, breast, ovarian, prostate, and colon cancer [9,10]. Chronic inflammation of tissues provides an environment that promotes cancer cell growth [9]. The vitamin D receptor (VDR) can target genes with roles in inflammation, cell growth, and cell differentiation [10]. There are polymorphisms of the VDR genes that can determine an individual’s susceptibility to a type of cancer. For example, the VDR Fok1 gene polymorphism increases the risk of oral cancer because apoptosis is reduced [10,43].

Vitamin D can reduce inflammation by regulating the inflammatory pathway [9]. This involves downregulating genes that initiate cancer, such as MAP kinase phosphatase 5 (MKP5), nuclear factor kappa B (NF-κB), and leukocytes [6,8]. These nutrigenomic effects make vitamin D effective at reducing the risk of leukemia, colorectal, breast, prostate, and pancreatic cancer [5,6,10]. In a Mendelian randomization study where four single nucleotide polymorphisms (RS2282679, RS10741657, RS12785878, and RS6013897) were associated with vitamin D, there was little evidence for a linear casual association between circulating vitamin D concentration and risk of various types of cancer [44]. Finally, recent studies have proven beyond a reasonable doubt that vitamin D obtained from food (fish, dairy, eggs, and mushrooms) can be metabolized and activated through a CYP11A1-driven non-canonical metabolite pathway, and its dysregulation promises new methods for vitamin D-based cancer therapies [7,43].

### 3.4. Vitamin E

Food sources rich in vitamin E (tocopherols) are nuts, plant seeds, and oils. Humans rely on these food sources to obtain vitamin E. Until the 20th century, many selenium and vitamin E cancer preventions (SELECT) and α-tocopherol and β-carotene cancer prevention (ATBC) trial studies focused on α isoform of vitamin E (α-tocopherol) with no meaningful results on its anticancer effects. A paradigm shift to other isoforms of tocopherols gave an insight into the signaling pathway regulated by vitamin E [29]. The mechanism by which vitamin E exerts its anticancer effects includes scavenging reactive nitrogen and oxygen species, anti-angiogenic effects, inhibition of 3-hydroxy-3-methylglutaryl coenzyme-A (HMG-CoA) reductase enzyme, and inhibition of the nuclear transcription factor (NF-kB) signaling pathway [30]. Other forms of vitamin E (β, δ, and ɣ) are important antioxidant vitamins. Similar to vitamin D, tocopherols also inhibit multiple pathways that promote cancer progression, such as COX and 5-lipoxygenase-catalyzed eicosanoids [8].

Of interest, vitamin E regulates and activates transcriptional factor 3 (sFAT3). This evidence strongly suggests that the forms of vitamin E can protect against cancer or act as an adjuvant for improving cancer therapy. The uniqueness of vitamin E is found in its chromanol ring and phytyl side chain. The saturated side chain known as tocotrienols with three double bonds on the side chain of vitamin E makes it capable of scavenging lipid peroxide radicals by donating hydrogen bonds, while the phenol group on the chromanol ring makes it a good antioxidant source by effectively quenching free radicals via one-electron reduction, thereby preventing the propagation of free radical reactions in lipid peroxidation [45]. In essence, little is still known about the anticancer properties of vitamin E and its precursors, which offers research opportunities for scientists to explore in the future [30].

### 3.5. Vitamin K

Vitamin K is necessary for blood clotting and the prevention of bleeding. It is found in leafy green vegetables, meats, dairy, and eggs. Limited evidence exists on the association between vitamin K and cancer. However, there is growing evidence that vitamin K is involved in tumorigenesis [23]. Refolo and colleagues postulated that vitamin K2 inhibits cancer cell proliferation in HepG2 and HLF human cell lines through downregulating PI3K/Akt signaling [31]. In the cancer and nutrition Heidelberg cohort study of 24,340 cancer-free patients, there was a significant inverse association between vitamin K2 intake and cancer mortality [46]. The mechanism of action of the signaling pathway of whether vitamin K is either upregulated or downregulated is not yet elucidated. However, the undercarboxylated form of prothrombin, a precursor of vitamin K, is upregulated in lowering the risk of HCC signaling [23,47]. More studies are needed to ascertain the hypothesis that vitamin K-related pathways can be used to diagnose, treat, and prognosticate a number of cancer-related diseases.

### 3.6. Vitamin B

There are eight B vitamins, each with specific functions in the body. B vitamins are one of the most important groups of vitamins owing to their direct impact on cell metabolism, brain functions, and energy levels. In general, they are necessary for the production of hormones and neurotransmitters, the breakdown of macronutrients, and immune function [48,49]. The latter functions suggest the current treatment methods that scientists are looking into for the management of cancer patients. The B vitamins can be obtained by consuming a variety of fruits, vegetables, and animal products [48,49] since most mammals cannot synthesize them on their own. Recent data showed that cancer patients are often deficient in vitamin B1, especially those undergoing chemotherapy; therefore, there is a signal of genetic alteration in cancer patients. Thiamine has effects on transcriptional activities of the master metabolic regulator and genome guardian p53, in which the direct target of genome guardian regulates cell cycle dynamics and DNA damage response. One mechanism of action of the correlation between vitamin B and cancer is related to a p53/p21-dependent change in the partitioning of glutamate conversion of 2-oxoglutarate through glutamate oxaloacetate transaminase (GOT2) or the glutamate dehydrogenase (GDH)-linked NAD(P)-dependent metabolism of 2-oxoglutarate in the affiliated thiamine pathway [24]. The study between vitamin B2 and breast cancer in a 2017 metastasis analysis involving 12,268 breast cancer human subjects showed a weak correlation, suggesting a low association with cancer therapy [25]. On the other hand, vitamin B3 is related to GPR109A activation, which functions as a tumor suppressor with effects on lipids and tissue-specific regulation of metabolism and inflammation [26], while vitamin B_6_ (pyridoxal phosphate) modulates the fate of sulfur and selenohomocysteine between transsulfuration and the transmethylation pathway in cellular metabolism under high oxidation states linked directly to cancer therapy in recent and modern research [26]. Furthermore, deficiencies of vitamin B_5_ are very rare but linked to inflammation and cancer. Uptake requires sodium-dependent multivitamin transporter (SMVT), the same transporter of vitamin B_7_ (biotin). More studies are, therefore, required to understand the role of vitamin B_7_ in cancer therapy. However, analysis of pantothenate shed light on the role of pantetheine in human health [27]. Moreover, elevated B_12_ has been linked to the development of cancer in two recent studies [19]. This is because vitamin B_12_ is a co-factor of methionine synthase implicated in methylation reaction as well as the synthesis of purine bases, which are crucial in tumor-initiating cells and cell proliferation. Although, the mechanism is poorly understood and more studies are required [19]. Finally, vitamin B_9_ is referred to as folate, the natural form present in foods, and folic acid, the synthetic form found in supplements and fortified food [5,6,7,10,12]. Similar to vitamin C, the effect depends on the amount consumed [7]. Low concentrations of folate in the blood are associated with double-stranded breaks in the DNA caused by the insertion of uracil, which can then cause carcinogenesis due to mutation [10]. Low folate is associated with colorectal, pancreas, prostate, or breast cancer [7]. However, high amounts of folate are associated with the formation of pre-cancerous cells as well [7,10,12]. This is because folate is a co-factor for enzymes involved in RNA and nucleotide synthesis by donating a methyl group [12]. When the amount of folate in the body is too high, increased methylation leads to increased carcinogenesis due to polymorphisms of the C4639T and serine hydroxymethyltransferase 1 (SHMT1) C1420T genes [10]. Therefore, it has been found that folic acid supplements are not necessary because North Americans receive enough from foods naturally containing folate and fortified foods (Araghi et al., 2019; Nasir et al., 2020). Overall, folate is effective at reducing the risk of gastric, colorectal, breast, and pancreatic cancer [5,6,10].

## 4. Trace Minerals and Cancer

Many trace minerals have been implicated in cancer. Some trace elements are positively linked to the pathogenesis of cancer, while others have a negative correlation and are even associated with lowering the risks of cancer. This is due to the activities and roles played by these trace elements in many cancer-regulating enzymes. Mercury, cadmium, and lead are the primary trace elements associated with an increased risk of cancer [50,51]. These elements are not biodegradable and will persist in the soil, air, and water, leading to the potential risk for bioaccumulation [52]. They can enter the body by inhalation, cutaneous absorption, or the gastrointestinal tract [52]. A major source of mercury and cadmium in food is fish and seafood, particularly large fish, such as yellowfin tuna and swordfish [7,51]. There is evidence that mercury increases the risk of breast cancer, as 55% of lobules analyzed from breast tissue after mastectomies contain mercury [50]. Additionally, lead contamination of vegetables is a growing global concern. Although the rates of contamination in developed countries have declined, there are developing countries with high rates of lead contamination in the soil, which therefore causes contamination of vegetables [53]. This is an increasing global concern as there is a high amount of global trade of food products [53]. In this review, we discuss the roles of cancer-lowering trace elements and their specific signaling pathways.

### 4.1. Selenium

Selenium is an ultra-trace mineral acquired in the diet [10]. The food highest in selenium is Brazil nuts, followed by organ meats and seafood [10]. There are three dietary forms of selenium: organic, inorganic, and selenium-containing nanoparticles (SeNPs), all of which have beneficial effects [13]. Selenium and selenium-containing compounds, such as the 25 selenoproteins found in humans, have multiple functions, including antioxidant, anti-inflammatory, apoptosis, improved immune response, carbohydrate metabolism, cardiovascular health, thyroid hormone regulation, and brain function [10,12,13,15]. A wide range of functions of selenium in the body makes it effective at reducing the risk of multiple cancer types, including leukemia, prostate, breast, lung, colorectal, bladder, uterine, and ovarian cancer [6,10]. The only known selenium-containing carcinogen is selenium sulfide, which is found in anti-dandruff shampoos [32]. Selenium inhibits the cytochrome P450 enzymes, the enzymes that produce DNA-damaging compounds, and activates p53 and Rb signaling genes, which increases antioxidant function, resulting in decreased DNA damage and inducing the apoptosis of cancerous cells [10,12,13].

Furthermore, selenium has a dual role in ROS, and this explains the reason for conflicting results often noticed in selenium cancer research [14]. In cancer therapy, organic selenium compounds have been linked to the epigenome of a cell through histone modifications, ncRNA expression, and DNA methylation, whose expression is regulated in various types of cancer, while inorganic selenium compounds, such as Se-methylselenocysteine (MSC), Selenite, SeMet, and Se-allylselenocysteine, could be effective at inhibiting the activities of histone deacetylases and DNA methyltransferase [14]. Epidemiologic and experimental evidence has indicated that selenium regulates cyclooxygenase-2 and extracellular signal-regulated kinase signaling pathways by activating AMP-activated protein kinase in colorectal cancers [10,12,13,15].

### 4.2. Zinc

The main food sources of zinc are mushrooms, legumes, whole grains, and meat [1,54]. Zinc is the most abundant trace element because it is required for the function of more than 3,000 transcription factors and is a co-factor for more than 300 enzymes and DNA repair proteins [15]. It has many functions, including cell proliferation, immune function, DNA repair, and antioxidant functions [12,15,33]. For example, the copper/zinc superoxide dismutase (CuZn-SOD) enzyme is an abundant antioxidant in cells [15]. Additionally, zinc acts on the p53 signaling gene to initiate DNA repair [15]. It is a co-factor for enzymes involved in DNA repair and is required for the removal of oxidized guanine [12]. There is little research regarding the specific types of cancers prevented by zinc because it has many effects on the body [15].

Recent studies showed that efficacious chemotherapy has not successfully treated or prevented prostate, liver, pancreatic, and other types of carcinomas, which exhibit decreased zinc in malignancy [15]. This suggests that there is a possible solution if more studies are carried out on zinc and zinc-containing cancer-regulating enzymes due to zinc ZIP transport downregulation in these forms of cancer. It has been shown that zinc deficiency in prostate cancer cells promotes cell survival through PI3K signaling pathway. PI3K is stimulated leading to the phosphorylation of protein kinase B (Akt) via phosphoinositide-dependent kinase (PDK). pAKT then phosphorylates the cyclin-dependent kinase inhibitors p21, thereby affecting the cell cycle activity of the cyclin D/cyclin-dependent kinase 4 (CDK4) complex. Therefore, zinc deficiency affects p21 signaling pathways, leading to cell proliferation in prostate cancer cells.

### 4.3. Other Required Minerals

Calcium is obtained from dairy, fish, leafy green vegetables, eggs, and nuts [55]. It has many essential functions in the body, such as heart, muscle, and gastrointestinal health; formation of bones; and synthesis of blood cells [55]. Calcium may reduce the risk of colorectal cancer by inhibiting the replication of precancerous cells [56]. Copper is found in the liver, seafood, nuts, seeds, and legumes [55]. It is required for many enzymes to function, such as cytochrome c oxidase, which is involved in energy formation [55]. There are many enzymes containing copper, which are known as cuproenzymes [6,13,17,24,57]. These enzymes have roles in respiration, pigmentation, iron transport, superoxide dismutation, and biosynthesis of the extracellular matrix [57]. Magnesium can be acquired from spinach, legumes, seeds, whole grains, nuts, and avocados [55]. It is a component of many enzymes, which have a variety of functions, such as ATP production, bone health, and modulating potassium-calcium channels in the heart muscle to modulate neuronal contraction [55]. Magnesium is also effective at reducing the risk of colorectal cancer due to its ability to reduce the incidence of polyps in the colon [22]. Manganese is found in whole grains, nuts, seeds, legumes, and leafy vegetables [55]. It is a co-factor for many enzymes, which allow for the production of connective tissue, and promotes blood clotting [17,33,55,58]. The main source of sodium is table salt [55,58]. Sodium is required for cellular transport, nutrient absorption, electrolyte balance, and nerve function. Although sodium has many health benefits, diets high in sodium can increase the risk of cancer, particularly stomach cancer [55,58]. Most trace elements like calcium (Ca), magnesium (Mg), manganese (Mn), and sodium (Na) are required by several enzymes for activities. These proteins are enriched in cancer-related pathways and pathways participating in immune responses, such as mitogen-activated protein kinase (MAPK) signaling pathways as well as complement and coagulating cascades.

## 5. Anti-Inflammatory Properties of Micronutrients in Relation to Cancer

**Figure 2:**
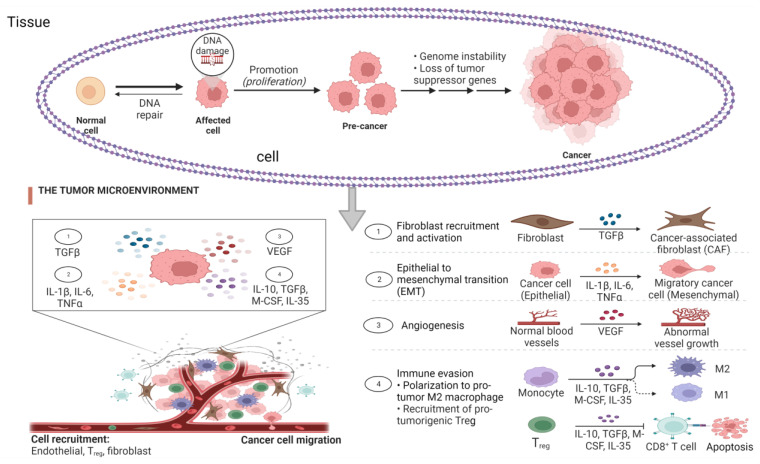
Stages of cancer and the role of inflammatory biomarkers. The tumor microenvironment (TME), comprising the fibroblasts and vascular cells as well as the inflammatory immune cells, is predominated by the macrophages after DNA damage without DNA repair with evidence of increased concentration of cytokines leading to cytokine storm. The presence of macrophages in the tumor tissue is positively correlated to the activation of cancer stages, which include cellular transformation, promotion, survival, proliferation, angiogenesis, and metastasis.

The role of inflammation in the development of cancer cannot be underestimated as it promotes all stages of tumorigenesis ranging from cellular transformation, promotion, survival, proliferation, angiogenesis, and metastasis [59]. Based on the recent findings, the anti-tumorigenic function of the immune system can be summarized as immunosurveillance and immunological sculpting of tumor heterogenicity since inflammation has a great impact on the composition of the tumor microenvironment (Table 2). The microenvironment comprises the fibroblasts and vascular cells as well as the inflammatory immune cells predominated by the macrophages [14,60]. Chronic inflammation is characterized by sustained tissue damage and has been found to mediate a wide range of diseases including cardiovascular diseases, diabetes, arthritis, pulmonary diseases, autoimmune disease, and even cancer [61]. The presence of lymphocytes in tumors provides a possible link between inflammation and cancer, suggesting that the ability to reduce inflammation in tumors and/or cancer can be a target for cancer management and treatment (Figure 2). However, anti-inflammatory agents must possess antioxidant properties. Therefore, anti-inflammatory agents can also be considered antioxidant agents. Most vitamins and micronutrients are known for their antioxidant activities, signifying their role of nutrigenomics in cancer therapy [62].

**Figure 3:**
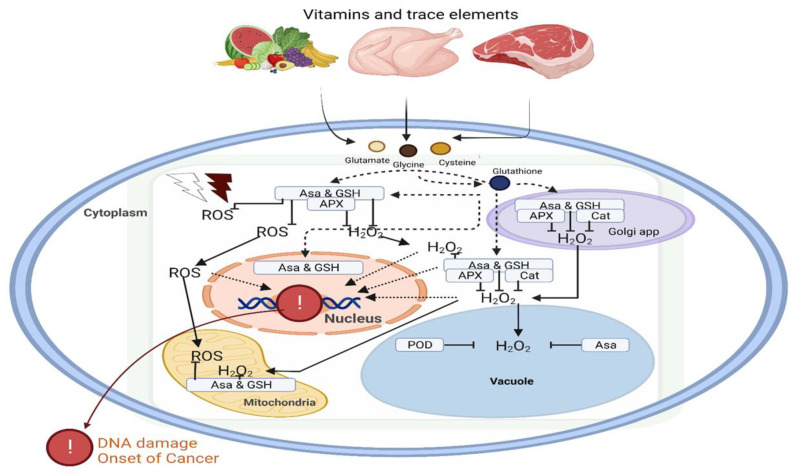
Dietary vitamins and minerals contribute to the antioxidant defense system. The impact of oxidative stress on DNA damage and initiation of carcinogenesis can be regulated by vitamins and trace elements in the different organelles of the cell. Vitamins such as ascorbic acid and reduced glutathione play a key role in oxidative homeostasis. Abbreviations: ROS, reactive oxygen species; APX, ascorbate peroxidase, H_2_O_2_, hydrogen peroxidase; POD, peroxidase; GSH, glutathione peroxidase; Asa; ascorbic acid.

Antioxidant agents tend to reduce the excessive production of ROS and the subsequent oxidative stress. They carry out these functions by preventing cellular damage, thereby reacting to and eliminating oxidizing free radicals to find relevance in adjuvant chemotherapy [63]. Moreover, several studies have suggested the combination of antioxidants in chemotherapy for specific cancer settings [64]. Cytotoxic cancer chemotherapeutic agents generate ROS. Therefore, the availability of vitamins to act as antioxidant agents (Figure 3) is important during the management and treatment of cancer to reduce the severity of ROS without interfering with the drug’s antineoplastic activity [65]. Since there are limited studies on the benefits and safety of antioxidant use during cancer treatment, this review attempts to emphasize the importance of future research targeting specific signaling pathways that are regulated by vitamins and micronutrients in the treatment and management of cancer.

**Table 2:** Anti-inflammatory and anticancer activities of micronutrients through regulation of specific cell signaling pathways.

Micronutrient	Signaling Pathway(s)	Types of Cancer	Anti-Inflammatory & Anti-Cancer Activity	References
Vitamin A	Activation of NF-κB, downregulation of the synthesis, and secretion of IgE through RARα	Chronic myelogenous leukemia, leukemia, cervical cancer, non-small cell lung cancer (NSCLC), head and neck cancer	Growth arrest, apoptosis, re-differentiation, halting of inflammatory cytokines	[35,66,67,68,69]
Vitamin B	Downregulation of the vascular endothelial growth factor (VEGF) signaling pathway	Lung, breast, and thyroid cancer; acute myeloid leukemia (AML); malignant lymphoma; acute lymphoblasyic leukemia; non-Hodgkin lymphoma; and melanoma	Maintenance of the VEGF levels and reduction in angiogenesis	[5,27,70]
Vitamin C	Activation of ten-eleven translocation proteins (TET) and downregulation of pluripotency factors	Primary or metastatic brain tumors, NSCLC, cervical cancer, and breast carcinoma (stage II)	Degradation of hypoxia-inducible factor (HIF-1); essential for tumor cell survival; activation of NKT cells and monocytes	[62,71,72,73]
Vitamin D	Activation of MAPK kinases, PI3 kinases, and phospholipase A2 and C signaling pathways (PI3K/AkT/ERK1/2/MAPK)	Skin cancer, melanoma, lymphoma, and leukemia	Inhibition of angiogenesis and metastasis; induction of apoptosis and autophagy	[9,74]
Vitamin E	Activation of NF-κB and downregulation of cyclooxygenase and 5-lipoxygenase-catalyzed eicosanoids	Head and neck cancer, esophageal cancer, hepatocellular cancer, AML, solid malignancies (lung, pvarian, rhinopharynx, urethral, gastric, testicular, ethmoidal, and tongue cancer), oral and uropharynx cancer.	Induction of autophagy and reduction of angiogenesis	[45]
Vitamin K	Downregulation of 12-lipoxygenase signaling pathway	solid malignancies (lung, pvarian, rhinopharynx, urethral, gastric, testicular, ethmoidal, and tongue cancer).	Differentiation and apoptosis as well as maintenance of cellular redox homeostasis	[75]
Selenium	Downregulation of Nrf2 signaling pathway	All types of cancer	Activation and proliferation of B cells and various enzyme activation	[76,77]
Zinc	Upregulation of NK cells	All types of cancer	Enzyme activation. Stabilization and regulation of cellular signaling	[78]
Copper	Regulation of Nrf2 signaling pathway	All types of cancer	Generation of free radicals (reactive oxygen and nitrogen species)	[79]
Magnesium	Activation of Stat3 and NF-κB signaling pathways	All types of cancer	Reduction of inflammatory biomarkers, such as C-reactive protein (CRP) and increasing nitric oxide (NO) levels	[80]
Manganese	Activation of NF-κB and hypoxia-inducible factor (HIF-1)	All types of cancer	Maintenance of superoxide dismutase (SOD) and production of reactive species	[81]

Abbreviations: NF-κB, nuclear factor kappa light-chain-enhancer of activated B cells; RARα, retinoic acid receptor alpha; NKT, natural killer T cells; MAPK, mitogen-activated protein kinase; AKT, protein kinase B; ERK, extracellular signal-regulated kinase; Nrf2, nuclear factor erythroid 2–related factor 2.

## 6. Mediterranean Diet and Nutraceuticals

Based on scientific evidence, the most commonly recommended diet that is effective at preventing cancer risk is the Mediterranean diet. The American Cancer Society (ACS) publishes the Diet and Physical Activity Guideline for physicians to make dietary recommendations to patients [9]. The diet recommendations in this publication are very similar to the Mediterranean diet. A change in diet is usually recommended as an early preventative measure because it is a risk factor that can be easily modified and reduces the risk of cancer by 30–50%. There are more than 80,000 new cancer cases per year in the USA that are associated with poor diet [3,37]. The Mediterranean diet has many variants but the common one includes foods with beneficial micronutrients and avoids foods associated with cancer, such as saturated fatty acids, trans-unsaturated fatty acids, high-sugar drinks, red meat, and processed meats. The diet consists of a regular intake of a variety of fruits and vegetables, high-fiber grains, moderate dairy intake, moderate alcohol intake, and low meat intake. This diet provides all the necessary antioxidant vitamins and micronutrients to reduce cancer risk [42]. Transcriptional response to a Mediterranean diet intervention has been found to exert a modulatory effect on neuroinflammation signaling pathways as well as the PI3K/Akt/MAPK/NF-κB/mTOR signaling pathways. Although the Agency for Research on Cancer (IARC) categorized alcohol as a carcinogen in 1988 due to its acetaldehyde content, some alcoholic drinks, such as red wine, contain polyphenols, which are beneficial antioxidants [41].

Recently, consumer’s interest in the prevention of cancer through dietary supplements has increased considerably, and scientific assessment of such nutraceutical formulation has started to appear. Exposure to endogenous and exogenous carcinogenic factors such as chemical agents and UVA/UVB radiation can induce DNA double-strand breaks (DSB) [82], and failure to effectively repair DNA DSB may lead to the development of cancer [83]. Activation of nuclear factor E2-related factor 2 (Nrf2), the master regulator transcription factor of antioxidant and cytoprotective genes, could be effective for supporting oxidative homeostasis and thus reducing the risk for chronic disorders including cancer [84,85]. Recently, apple peel polyphenols, particularly flavonoids, have been shown as strong dietary antioxidants, which suppress carcinogen-induced DNA damage in human bronchial epithelial (BEAS-2B) cells in the healthy state [86]. Apple flavonoid-pretreated cells showed lower cytotoxicity, intracellular ROS, and DNA fragmentation. Apple flavonoids also facilitated the phosphorylation of DNA-dependent protein kinases (DNA-PK) and thus the initiation of DNA damage repair (DDR) mechanisms. Many studies have confirmed that a diet rich in flavonoids plays a role in genome stability and reduces cancer risk and mortality [83,85,87]. An antioxidant formulation that comprises antioxidant vitamins (ascorbic acid, folate) and the vitamin A precursor β-carotene with or without the combination of apple flavonoids has alleviated DNA damage in carcinogen-induced bronchial epithelial BEAS-2B cells through the activation of the ATR/Chk1 signaling pathway [88]. Similarly, an antioxidant formulation of vitamin C, vitamin B_9_, vitamin B_12_, vitamin E, α-lipoic acid, coenzyme Q10, astaxanthin, zeaxanthin, quercetin, and sodium selenite ameliorated DNA damage induced by γ-irradiation in BEAS-2B cells [89]. The dietary antioxidant mixture significantly reduced the induction of the tumor suppressor protein p53 and DNA damage-associated γ-H2AX phosphorylation. Similarly, the vitamin C and flavonoid-rich ayurvedic herb mixture, Triphala, holds the potential to prevent and treat oral cancer [90]. The above scientific evidence suggests that therapeutic supplements can be developed using antioxidant vitamins, other micronutrients such as minerals, and phytonutrients to reduce the risk of cancer due to exposure to carcinogenic environmental factors, genotoxic chemotherapy, or diagnostic radiation such as X-rays.

## 7. Conclusions

Reports from recent studies suggest that the novel treatment of cancer and cancer-related diseases lies partially in diet and its modifications. The applications of nutrigenomics could be a promising approach for preventing or reducing the risk for cancer through dietary modifications and enhancing chemotherapy. The mechanisms by which these vitamins and trace elements assist in nutrigenomics are partly through the regulation of PI3K/Akt/MAPK/NF-κB signaling pathways. Further study of how micronutrients affect specific genes would be beneficial in reducing cancer cases globally. The identification of these specific genes, and the related nutrigenomics interactions, through human genome sequences may improve the stratification of cancer risk on a population and individual level. Further research is required to identify the specific genes, biochemical pathways, and cell signaling that are affected by certain nutrients and how the adverse impacts can be regulated. Micronutrients, such as vitamin A, vitamin C, vitamin D, folate, selenium, and zinc, are essential dietary constituents for cancer prevention. These micronutrients have a common function as antioxidant and anti-inflammatory agents; however, they also have specific functions in regulating genes associated with carcinogen metabolism and carcinogenesis. This emphasizes the importance of reducing DNA damage by ROS to prevent cancer development. Overall, the Mediterranean diet is a good example of a diet that includes many cancer-preventative antioxidant vitamins, other micronutrients, and phytochemical food bioactives.

## Figures and Tables

**Table 1:** Summary of the nutrigenomic effects of micronutrients on the potential for prevention of cancer.

Micronutrient	Food Sources	Genes Affected	Beneficial Effects	Preventable Cancers	References
Vitamin A	Liver, fish oils, eggs, milk, leafy green vegetables	JAK-STAT ^1^, RARs ^2^, RXRs ^3^, JNK ^4^, Pakt/pERK/Pegfr-genes	Antioxidant, apoptosis, immune response	Gliomas, lung, prostate, colon, breast	[2,5,6,10,11,15]
Vitamin B_1_ (Thiamine)	Pork, fish, legumes, yogurt, sunflower seeds	p53/p21 affiliated 2-oxoglutarate thiamine pathway	Cell upregulation	All types of cancer	[24]
Vitamin B_2_ (Riboflavin)	Meat, fish, dairy products, eggs	Weakly studied	Cell upregulation targeted	Breast	[25]
Vitamin B_3_ (Niacin)	Liver, meat, fish	GPR109A activation. Tumor suppression	Cell upregulation and inflammation	All types of cancer	[26]
Vitamin B_5_ (Pantothenic acid)	Organ meats, chicken, mushrooms, nuts, seeds, milk	Sodium-dependent multivitamin transporter (SMVT) pathway, pantothenate/pantetheine pathway	Cell upregulation	Solid cancer	[27]
Vitamin B_6_ (Pyridoxine)	Liver, poultry, fish, chickpeas, dark green leafy vegetables, bananas, oranges	Sulfur and selenohomocysteine transsulfuration and transmethylation pathway	Cellular metabolism under high oxidation states	All types of cancer	[28]
Vitamin B_7_ (Biotin)	Liver, fish, eggs, avocados, sweet potato, nuts, and seeds	Sodium-dependent multivitamin transporter (SMVT) pathway, pantothenate/pantetheine pathway	Cell upregulation	Solid cancer	[27]
Vitamin B_9_ (Folate)	Liver, seafood, beans, sunflower seeds, dark green leafy vegetables, fruits	C4639T, SHMTI C1420T ^9^	Apoptosis, anti-inflammatory	Colon, breast, prostate, pancreatic, cervical	[5,6,9,10]
Vitamin B_12_ (Cobalamin)	Meat, fish, eggs, dairy	Methionine synthase pathway	Apoptosis and cell anti-proliferation	All cancer types	[19]
Vitamin C	Fruits and vegetables	Bcl-2 ^5^, 2-oxoglutarate-dependent dioxygenase pathway, ten-eleven translocase (TET) DNA demethylase pathway	Antioxidant, apoptosis, immune response, prevent carcinogen formation, p53 upregulation, decreased ATP levels, suppression of antioxidant gene expression of NRFα	Solid tumours, malignancies	[6,10]
Vitamin D	Fish, fortified milk and orange juice	VDR Fok1 ^6^, MKP5 ^7^, NF-κB ^8^ Dysregulation of wnt/β-catenin metastasis	Apoptosis, anti-inflammatory, cell–cell adherence	Colon, prostate, leukemia, skin	[5,6,9]
Vitamin E	Peanuts, almonds, sunflower seeds and oil, pumpkin	Transcriptional factor 3 (sFAT3), NF-kβ pathway, COX2 pathway, 5-lipoxygenase-catalyzed eicosanoids pathways	Apoptosis, anti-inflammatory	All cancer types	[29,30]
Vitamin K	green leafy vegetables	CYP11A1-driven non-canonical metabolite pathway	Apoptosis, anti-inflammatory, cell–cell adherence	All carcinomas	[31]
Selenium	Organ meats, seafood, Brazil nuts	p53, Rb ^10^, DNA methyltransferase pathway, histone deacetylase pathway	Antioxidant, apoptosis, immune response, anti-inflammatory	Leukemia, prostate, lung, colorectal, bladder, uterine, ovarian	[10,12,13,14,15,32]
Zinc	Red meat, poultry, milk, beans, nuts	Component of many transcription factors and enzymes	Antioxidant, p53 ^4^, immune function	All cancer types	[12,15,33]
Copper	Organ meats, shellfish, seeds, nuts	Component of many transcription factors and enzymes	Antioxidant, apoptosis, immune response, anti-inflammatory	All cancer types	[12,15,33]
Magnesium	Legume dark green leafy vegetables, nuts, seeds	Component of many transcription factors and enzymes	Antioxidant, apoptosis, immune response, anti-inflammatory	All cancer types	[12,15,33]
Manganese	Shellfish, nuts, soybeans, black pepper, coffee, tea	Component of many transcription factors and enzymes	Antioxidant, apoptosis, immune response, anti-inflammatory	All cancer types	[6,10,12,13,14,15,32]

Abbreviations: ^1^ Janus kinase signal transducer and activator of transcription, ^2^ Retinoic acid receptor, ^3^ Retinoid X receptor, ^4^ c-Jun N-terminal kinases, ^5^ B-cell lymphoma 2, ^6^ Vitamin D receptor Fok1 polymorphism, ^7^ Mitogen-activated protein kinase, ^8^ Nuclear factor kappa B, ^9^ Serine hydroxymethyltransferase 1, ^10^ Retinoblastoma.

## Data Availability

No new data were created or analyzed in this study. Data sharing is not applicable to this article.
